# Efficacy of endovascular treatment for patients with acute large vessel occlusion stroke from the Western Sichuan Plateau and machine learning prediction models: a prospective study protocol

**DOI:** 10.3389/fneur.2025.1665032

**Published:** 2025-09-03

**Authors:** Chuanxi Duan, Yu Hu, Yuding Luo, Jiali Zhang, Pingchuan Liu, Junhao Li, Hai Xiong, Mengling Duan, Yupeng Niu, Ke Huang, Zhao Chen, Jian Wang

**Affiliations:** ^1^North Sichuan Medical College, Nanchong, China; ^2^Department of Neurology, The Affiliated Hospital, Southwest Medical University, Luzhou, China; ^3^Department of Neurology, Ya'an People's Hospital, Ya'an, China; ^4^Sichuan Agricultural University, Ya'an, China

**Keywords:** acute ischemic stroke, study protocol, high-altitude, endovascular treatment, cohort study, machine-learning

## Abstract

**Objectives:**

Stroke is the second leading cause of death and the third leading cause of disability among non-communicable diseases globally. The prevalence, incidence, and mortality rates of stroke are higher in high-altitude regions compared to lowland areas. However, compared to plain areas, the efficacy of endovascular therapy for large vessel occlusive acute ischemic stroke (LVO-AIS) in high-altitude regions remains unclear.

**Methods and Design:**

This study is a multicenter, prospective, endpoint-blinded cohort study. From January 2025 to December 2027, a total of 1,052 patients with acute large vessel occlusion ischemic stroke (LVO-AIS) from the Western Sichuan Plateau will be prospectively enrolled, including those who receive endovascular treatment and those who do not. Baseline characteristics and endovascular treatment details will be documented. Treatment decisions are guided by clinical practice guidelines, taking into account high-altitude real-world constraints such as patient or proxy refusal and delays in interhospital transfer. Medical records will be established for each patient, and a 180-day follow-up will be conducted. The primary outcome was the proportion of patients achieving functional independence [modified Rankin scale (mRS) range from 0 to 2] at 90 days. The secondary outcomes included the mRS score at 90 days, early neurological improvement rate [defined as a National Institutes of Health Stroke Scale (NIHSS) score of 0–2 or a reduction of ≥8 points from baseline within 24 h of enrollment], changes in NIHSS scores between day 7 ± 1 or discharge and baseline, quality of life as assessed by the five-level EuroQol five-dimensional questionnaire at 90 days, and cognitive function at 180 days will be assessed using mini-mental state examination and montreal cognitive assessment scores. Imaging outcomes will include the rate of successful reperfusion (defined as a modified Thrombolysis in Cerebral Infarction score ≥2b) and infarct volume measured within 5–7 days. Statistical analysis and fused optimized multimodal learning were blinded to the group assignments.

**Conclusion:**

This study aims to evaluate the efficacy of endovascular treatment compared with standard medical therapy in patients with LVO-AIS in the Western Sichuan Plateau and to develop an artificial intelligence–based prognostic model to refine treatment strategies for this and other high-altitude regions.

**Clinical trial registration:**

https://www.chictr.org.cn/showproj.html?proj=241870, ChiCTR2400092762.

## 1 Introduction

Stroke is a leading cause of death and disability globally, with large vessel occlusive acute ischemic stroke (LVO-AIS) posing a serious threat to health burden due to its high rates of disability and mortality. LVO-AIS (1) is a critical medical emergency characterized by acute occlusion of major cerebral arteries, such as the internal carotid artery or middle cerebral artery, leading to local cerebral ischemia and hypoxia which result in extensive cerebral infarction and severe disability or death if blood flow is not restored promptly. With the advancement of medical technologies, endovascular treatment (EVT) has become a crucial therapeutic approach for LVO-AIS, marking a new era in the treatment of ischemic penumbra by combining imaging techniques to identify low perfusion areas. It significantly improves reperfusion rates and enhances patients' neurological outcomes particularly when administered within 6 h of onset (2). The time window for thrombectomy in anterior circulation large vessel occlusion has now been extended to 24 h (3, 4). The Basilar Artery Occlusion Chinese Endovascular Trial (BAOCHE) (5) and Trial of Endovascular Treatment of Acute Basilar-Artery Occlusion (ATTENTION) (6) has confirmed the superiority of thrombectomy in the posterior circulation within 24 h.

An increasing number of individuals are engaging in tourism and recreational activities in high-altitude regions. Additionally, certain groups—including military personnel, miners, tour guides, and local residents—are chronically exposed to such environments. A global survey in 2021 estimated that approximately 500 million people reside at altitudes of ≥1,500 m (7), with China possessing the world's largest plateau region, home to roughly 12 million permanent residents (8). Notably, our previous literature review demonstrated that patients with ischemic stroke in high-altitude areas have significantly poorer functional outcomes compared with those in low-altitude regions. Moreover, differences in infarct localization have been observed, with anterior circulation strokes being more prevalent, and, according to TOAST classification, a higher incidence of large-artery atherosclerosis (9). These findings underscore the need for high-quality research to better inform clinical decision-making for ischemic stroke in high-altitude populations.

In the Western Sichuan Plateau, due to its unique geographical and climatic conditions, the incidence and mortality rates of stroke may be higher than in lowland areas, potentially linked to factors such as the hypoxic environment, cold climate, and lifestyle habits of the residents. Our previous meta-analysis (10) showed that the stroke prevalence is higher in high-altitude regions, with variations observed across different sampling types and geographical locations. Age, female gender, hypertension, and obesity are identified as potential risk factors in this population. However, it remains unclear whether patients with AIS in high-altitude areas benefit from EVT.

In recent years, the rapid development of machine learning is expected to enhance the predictive accuracy of the prognosis for certain treatment methods. The key advantage of the fused optimized multimodal learning model lies in its ability to handle imbalanced information across modalities. Progressive fusion effectively retains fine-grained information from earlier layers by gradually integrating features, while avoiding the information loss and feature conflicts that often arise in traditional early fusion methods (11). Orthogonal sequential fusion sequentially weights each modality's data, reducing inter-modal interference and ensuring the independence and diversity of each modality (12). Balanced multimodal learning dynamically adjusts the learning strategies for each modality, addressing the challenge of uneven contributions and improving the model's performance in complex multi-task learning scenarios (13). Together, these methods optimize the model's robustness in the presence of data imbalance, noise interference, and high-dimensional feature spaces, making it particularly suitable for high-complexity tasks such as medical imaging and genomics data analysis.

This study aims to prospectively collect data from LVO-AIS patients in the Western Sichuan Plateau to explore the efficacy of EVT in this population, providing evidence for endovascular interventions for AIS patients in the region. Furthermore, after validating the effectiveness of EVT for LVO-AIS in the Western Sichuan Plateau, an artificial intelligence-based prognostic prediction model will be developed using relevant data, significantly facilitating the treatment of AIS patients in this region.

## 2 Methods

### 2.1 Study design

**Figure 1:**
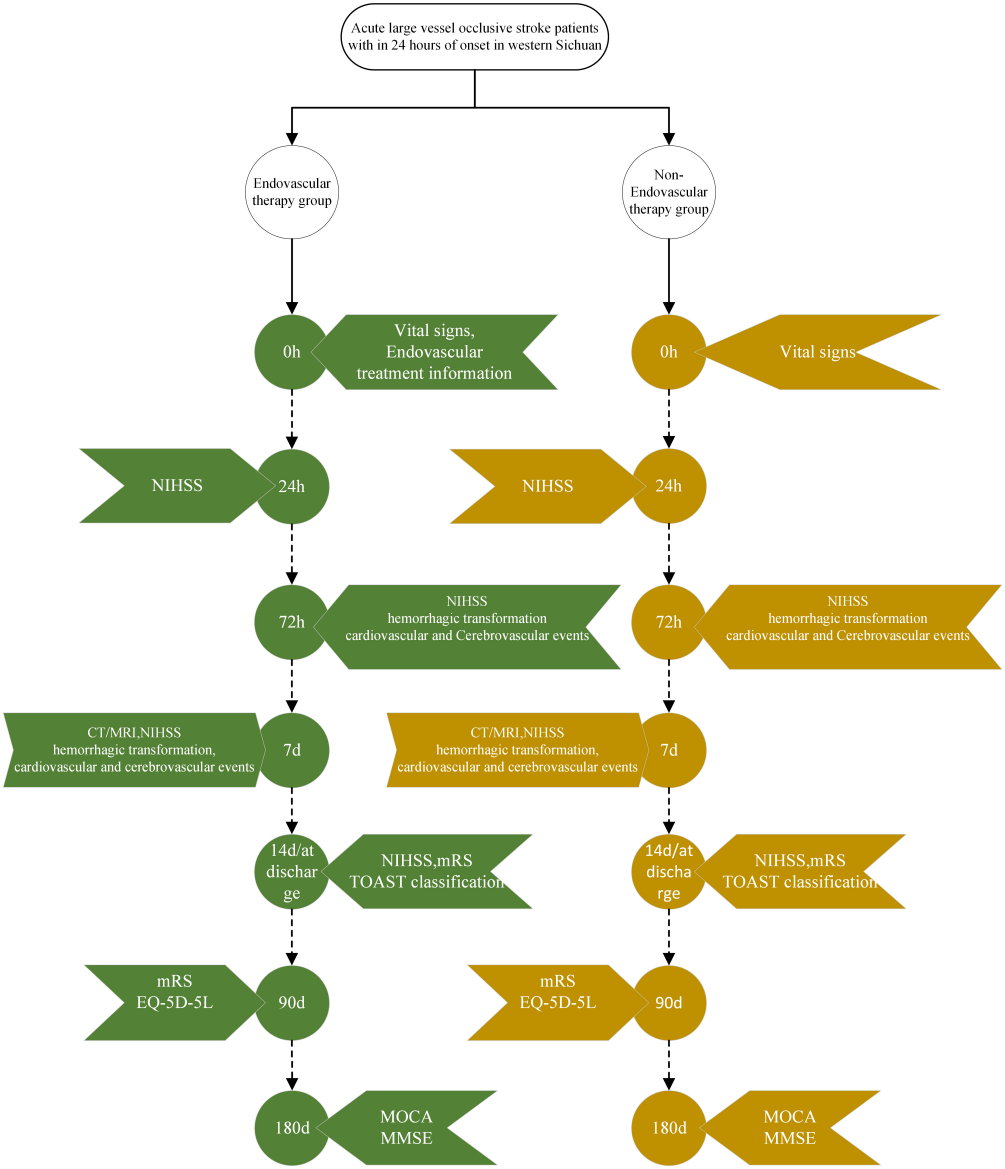
Research workflow diagram. NIHSS, National Institutes of Health Stroke Scale; CT, computed tomograph; MRI, magnetic resonance imaging; mRS, modified rankin scale; TOAST, trial of org 10,172 in acute stroke treatment; EQ-5D-5L, five-level EuroQol five-dimensional questionnaire; MOCA, montreal cognitive assessment; MMSE, mini-mental state examination.

This is a multicenter, prospective, endpoint-blinded study aimed at evaluating the efficacy of EVT in patients with LVO-AIS from the Western Sichuan Plateau, in comparison with standard medical treatment (SMT). We prospectively enroll LVO-AIS patients from this region who receive either EVT or SMT, collect baseline characteristics and EVT-related data, and conduct univariate and multivariate analyses to explore associated factors. As a prospective observational study, the exposure is not subject to artificial assignment or manipulation between the EVT and non-EVT groups. The follow-up period is standardized to 180 ± 7 days. Patients who failed to complete the follow-up were excluded to ensure data integrity. The detailed procedure is illustrated in Figure 1.

This study has been prospectively registered with the Chinese Clinical Trial Registry [registration number ChiCTR2400092762 (https://www.chictr.org.cn/showproj.html?proj=24187)], and it has been approved by the Ethics Committee of Ya'an People's Hospital (approval No. 2024045).

### 2.2 Sample size estimation and participants

We estimated that group sample sizes of 526 in each group will achieve 90.031% power to detect a difference between the group proportions of functional independence [modified Rankin scale (mRS) range from 0 to 2] at 90 days of 0.135. According to the Multicenter Randomized CLinical trial of Endovascular treatment for Acute ischemic stroke in the Netherlands (MR CLEAN) (14) trial, the proportion in control group is assumed to be 0.191 under the null hypothesis and 0.326 under the alternative hypothesis. The significance level of the test is 0.05. Considering a 20% dropout rate, the final sample sizes for both groups were 526. Given the large data requirements of machine learning, we doubled the sample size of each group based on the original estimates.

**Figure 2:**
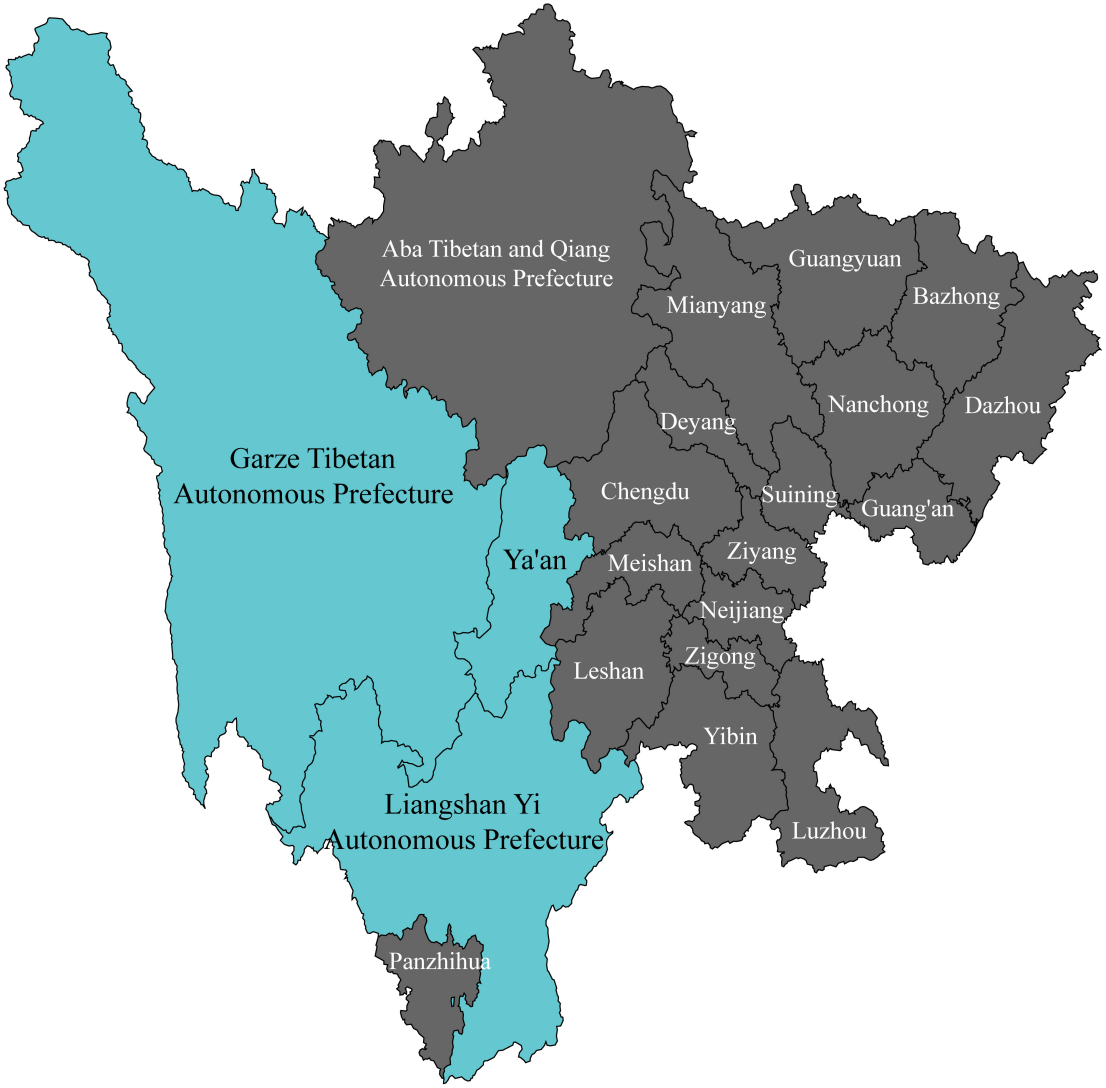
Recruitment coverage for study participants.

A total of 1,052 patients with acute large vessel occlusion stroke were enrolled from Ya'an People's Hospital, Ganzi Tibetan Autonomous Prefecture People's Hospital, and Liangshan Yi Autonomous Prefecture First People's Hospital (Figure 2), including both those who underwent endovascular treatment and those who did not. Although all patients met the current guideline-based indications for mechanical thrombectomy, some did not receive the procedure due to various factors, such as declining treatment over concerns about surgical risk or experiencing delays in transfer to thrombectomy-capable centers. In this study, these branch centers primarily served as referral sites or admitted patients who declined endovascular therapy. All participating centers implemented a standardized, homogeneous protocol for medical management. Prior to group assignment, patients who met the criteria for intravenous thrombolysis and had no contraindications were eligible to receive intravenous thrombolysis.

The inclusion criteria encompass: (1) aged above 18 years; (2) resides in a high-altitude region of Western Sichuan (with an altitude above 1,500 meters); (3) clinically diagnosed with AIS and confirmed by computed tomography angiography (CTA) or digital substraction angiography (DSA) to have large vessel occlusion; (4) The interval between last known well and enrollment will be within 24 h, and (5) signed informed consent and able to comply with follow-up requirements. The main exclusion criteria mainly include: (1) Pre-onset mRS score > 2; (2) computed tomography (CT) or magnetic resonance imaging (MRI) confirms the presence of intracranial hemorrhage or the infarct size exceeds one-third of the supply area of the middle cerebral artery; (3) History, previous imaging, or clinical judgment suggests the presence of a brain tumor, arteriovenous malformation, or intracranial artery dissection, or (4) History of head trauma within the past 3 months. Detailed exclusion and withdrawal criteria are provided in Appendix.

### 2.3 Outcomes

The primary outcome is the proportion of patients achieving functional independence (mRS 0–2) at 90 days post-stroke. The secondary outcomes are divided into clinical efficacy outcomes and imaging markers. The clinical efficacy outcomes include: (1) mRS score at 90 days; (2) early neurological improvement rate (defined as NIHSS score of 0–2 or a reduction of ≥8 points from baseline within 24 h of enrollment); (3) changes in NIHSS scores between day 7 ± 1 or discharge and baseline; (4) Quality of life assessed by five-level EuroQol five-dimensional questionnaire (EQ-5D-5L) at 90 days, and (5) Montreal cognitive assessment (MOCA) and mini-mental state examination (MMSE) scores at 180 days. The imaging endpoints include: (1) reperfusion rate [modified thrombolysis in cerebral infarction (mTICI) ≥ 2b on DSA] and (2) infarct volume based on diffusion-weighted MRI within 5–7 days. All data will be collected through electronic media and uploaded in real-time to a centralized data management platform for storage.

### 2.4 Statistical analysis

Statistical analyses will be conducted using R version 4.3.3 by an independent statistician blinded to group assignments. Continuous variables will be tested for normality using the Shapiro–Wilk test (α = 0.05). Normally distributed data will be presented as means ± standard deviations and compared using the independent *t*-test or one-way analysis of variance. Non-normally distributed data will be presented as medians with interquartile ranges and compared using the Wilcoxon rank-sum test or Kruskal–Wallis test. Categorical variables will be summarized as counts and percentages, with group comparisons performed using the χ^2^ test or Fisher's exact test.

Given the non-randomized allocation of treatment (EVT vs. SMT), potential confounding by indication will be addressed using propensity score methods. A logistic regression model including baseline covariates (age, sex, baseline NIHSS, onset-to-enrollment time, comorbidities, and relevant imaging findings) will be used to estimate the propensity score for receiving EVT. We will perform both propensity score matching (1:1 nearest-neighbor without replacement, caliper = 0.2 SD of the logit) and inverse probability of treatment weighting (IPTW) to adjust for baseline imbalances. Standardized mean differences (SMDs) will be reported to assess covariate balance before and after adjustment.

Primary analyses will be performed on the matched cohort, with IPTW analyses conducted as sensitivity analyses. Logistic regression will be used for binary outcomes, linear regression for continuous outcomes, and ordinal logistic regression for ordinal outcomes, all adjusted for residual imbalances after matching. Results will be reported as adjusted odds ratios (aOR) with 95% confidence intervals (CIs).

To address missing data, multiple imputation using chained equations (MICE) will be employed under the missing-at-random assumption, generating 20 imputed datasets. Analyses will be conducted on each dataset, and results will be pooled using Rubin's rules. Sensitivity analyses will compare complete-case and imputed results to assess robustness.

All hypothesis tests will be two-sided, with *p* ≤ 0.05 considered statistically significant. No interim analyses are planned.

### 2.5 Machine learning model

This study proposes a novel multimodal learning model—fused optimized multimodal learning (FOML)—designed to enhance the effectiveness of multimodal data fusion through progressive fusion, orthogonal weighting, and dynamic balancing mechanisms. First, the progressive fusion method retains the advantages of deep fusion while avoiding the feature loss issues associated with traditional early fusion approaches. By gradually integrating information across different layers, this method ensures that shallow-level features guide deeper fusion processes. This approach is expected to perform exceptionally well in future medical image analysis, particularly in scenarios combining imaging and clinical data, where diagnostic accuracy and model generalization capabilities are likely to see significant improvement (11). Secondly, the orthogonal sequential fusion (OSF) strategy sequentially weights and fuses data from each modality, using an adaptive mechanism to reduce inter-modal interference and enhance the unique information extraction capability of each modality. When dealing with data from modalities with significant dimensional differences, such as CT images, MRI scans, and genomic data, the OSF strategy can effectively improve the model's accuracy (12). Finally, the balanced multimodal learning framework addresses the issue of uneven contributions across modalities by dynamically adjusting the learning rate for each modality. In particular, in multi-task learning scenarios, it optimizes the objective function for each modality, enhancing overall task performance and preventing any single modality from exerting excessive influence (13). The method ensures the balanced integration of multimodal data, including images, genomic, and clinical information, in medical image classification tasks, thereby improving diagnostic efficiency and robustness. The FOML model combines three approaches: through the progressive layered fusion, orthogonal weighted fusion, and dynamic balancing mechanisms, not only enhances model accuracy but also strengthens its adaptability to noise and complex data, achieving multi-level integration. Additionally, considering that FOML operates at the task-wise in meta-learning, we implement a three-stage task division: meta-training, meta-validation, and meta-testing. Tasks are grouped and partitioned by domain/subject/scene/institution/time period to ensure mutual exclusivity among the three stages. To mitigate domain transfer overfitting risk, we apply Leave-One-Domain-Out (LODO) cross-validation, sequentially designating each domain as meta-test while using the remaining domains for meta-training and meta-validation, reporting cross-domain averages and variances. All inner-loop updates are constrained by a fixed number of steps and incorporate weight decay and gradient clipping; in cases of class imbalance, task-wise resampling or cost-sensitive loss functions are employed. Final reported metrics and interval estimates (via bootstrap or Bayesian hierarchical models) pertain solely to meta-test tasks, with explicit exclusion of hyperparameter tuning or reuse based on meta-validation tasks.

## 3 Discussion

This study aims to explore whether LVO-AIS patients in the Western Sichuan Plateau can benefit from endovascular treatment. Given the challenges many countries face with an aging population and the increasing burden of cerebrovascular diseases, research on stroke in plain areas has been extensive. In contrast, stroke-related research in high-altitude regions is relatively scarce. This study may offer some hope for clinical treatment of AIS patients in the Western Sichuan Plateau and similar regions, helping to alleviate stroke-related issues. However, as this study only includes LVO-AIS patient data from the Western Sichuan Plateau, its findings may provide treatment recommendations specifically for clinical practice and decision-making in high-altitude areas of China. The generalizability of these results to other high-altitude regions worldwide remains to be validated. Furthermore, as a follow-up study, it is inevitable to encounter loss of follow-up bias. In addition to conducting telephone interviews and follow-up visits upon returning to the hospital, we also attempted to conduct multidimensional follow-up through WeChat survey mini-programs to reduce bias. In summary, potential biases will be minimized through standardized protocols and adjustments for baseline parameters, ensuring the robustness and validity of the study findings.

The FOML model addresses common challenges in multimodal data fusion, particularly in complex applications such as medical image analysis, genomics research, and multi-task learning. In these domains, integrating data from diverse sources (e.g., images, gene expression, and clinical data) often requires managing heterogeneity, including differences in features, distributions, and noise. Traditional multimodal learning approaches often overlook modal differences or fail to effectively balance the contributions of each modality, limiting model performance. To tackle this issue, the FOML model employs a combination of progressive fusion, orthogonal sequential fusion, and dynamic balancing mechanisms. This approach allows for finer-grained adjustment of the fusion process, maximizing the contribution of each modality to the final prediction.

The key advantage of the FOML model lies in its ability to handle imbalanced information across modalities. Progressive fusion effectively retains fine-grained information from earlier layers by gradually integrating features, while avoiding the information loss and feature conflicts common in traditional early fusion methods. Orthogonal sequential fusion applies weighted fusion to each modality in sequence, reducing inter-modal interference and preserving the independence and diversity of each modality. Balanced multimodal learning dynamically adjusts learning strategies for each modality, addressing the challenge of uneven contributions and enhancing model performance in complex multi-task learning scenarios. Together, these techniques optimize the model's robustness in the face of data imbalance, noise interference, and high-dimensional feature spaces, making it particularly suitable for high-complexity tasks such as medical imaging and genomics data analysis.

In terms of experimental design, we will validate the FOML model on multiple multimodal medical datasets, encompassing various data types such as image data, gene expression data, and clinical data. To assess the performance of the FOML model compared to traditional fusion methods (e.g., progressive fusion, orthogonal sequential fusion, and balanced multimodal learning) in handling different tasks and data types, we will employ standard statistical analysis methods, including paired *t*-tests, analysis of variance, and Kruskal-Wallis tests, to ensure the statistical significance of performance differences between models. We will pay particular attention to the model's performance in the presence of noise and modality imbalance. We expect the FOML model to demonstrate higher accuracy and robustness under these challenges compared to traditional methods, further validating its superiority and potential applications in multimodal learning tasks.
