# Supplementary material for: Efficacy of endovascular treatment for patients with acute large vessel occlusion stroke from the Western Sichuan Plateau and machine learning prediction models: a prospective study protocol
Source: Front Neurol. 2025 Sep 3;16:1665032. doi: 10.3389/fneur.2025.1665032 (PMC12441512; doi:10.3389/fneur.2025.1665032)
Supplement: Supplementary file 2 [file Data_Sheet_2.PDF]

[Inclusion criteria]

The participants must meet all of the following inclusion criteria:

1. Aged 18 years or older;
2. resides in a high-altitude region of Western Sichuan (with an altitude above 1500 meters);
3. clinically diagnosed with AIS and confirmed by computed tomography angiography (CTA) or digital substraction angiography (DSA) to have large vessel occlusion;
4. The interval between last known well and enrollment will be within 24 hours;
5. signed informed consent and able to comply with follow-up requirements.

[Exclusion criteria]

Participants will be excluded from the study if they meet any of the following criteria:

1. Pre-onset mRS score > 2;
2. computed tomography (CT) or magnetic resonance imaging (MRI) confirms the presence of intracranial hemorrhage or the infarct size exceeds one-third of the supply area of the middle cerebral artery;
3. History, previous imaging, or clinical judgment suggests the presence of a brain tumor, arteriovenous malformation, or intracranial artery dissection;
4. History of head trauma within the past 3 months;
5. Presence of severe cardiovascular diseases, respiratory diseases, endocrine disorders, chronic illnesses, malignancies, or a life expectancy of less than 6 months;
6. Platelet count $< 40 \times 10^9/L$, recent active bleeding or bleeding tendencies within the past month, APTT/PT elevated > 2 times the normal value, or INR > 3.0 while using anticoagulants;
7. Pregnant or breastfeeding women;
8. Severe psychiatric disorders or cognitive impairments that prevent cooperation with the study;
9. Participation in other clinical trials;
10. Any other conditions deemed inappropriate for the study by the investigators.

[Criteria for Withdrawal]

Participants may withdraw from the study at any time without providing a reason, and their medical rights will not be affected. The investigators may also decide to discontinue a participant's involvement based on medical considerations. For participants who withdraw, the reason for withdrawal will be documented, and efforts will be made to complete the mRS score prior to withdrawal.
